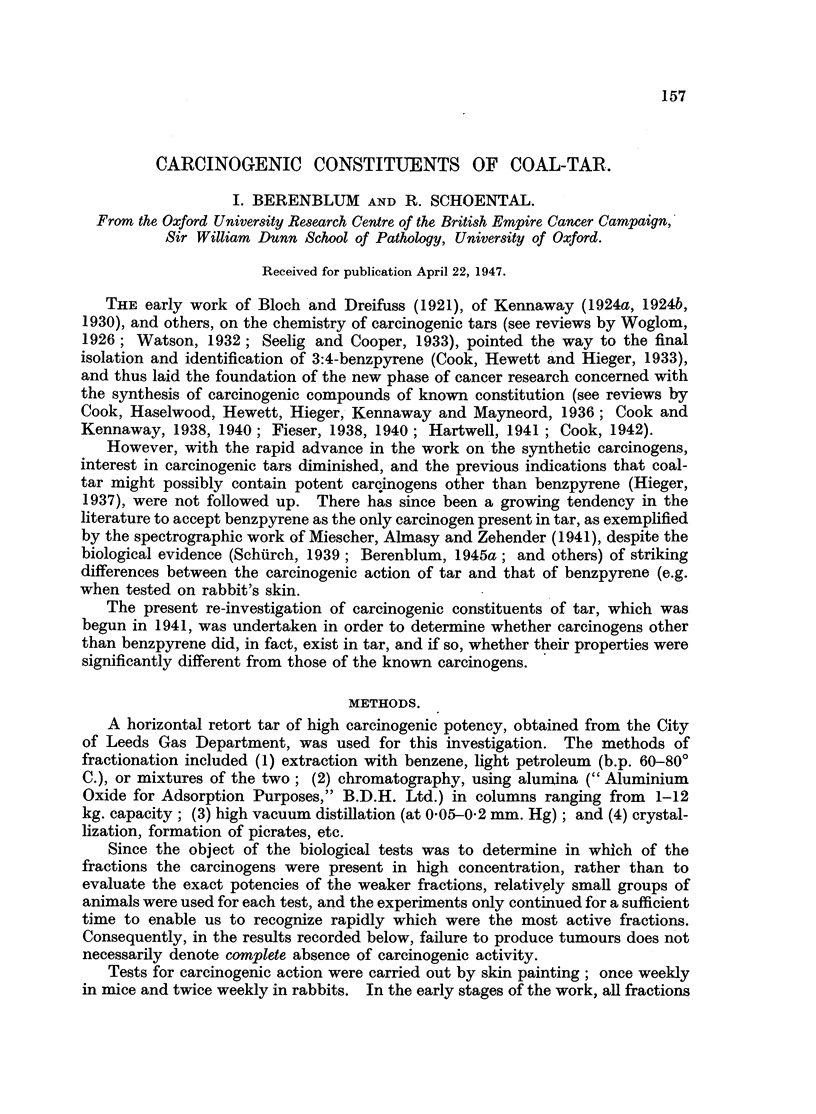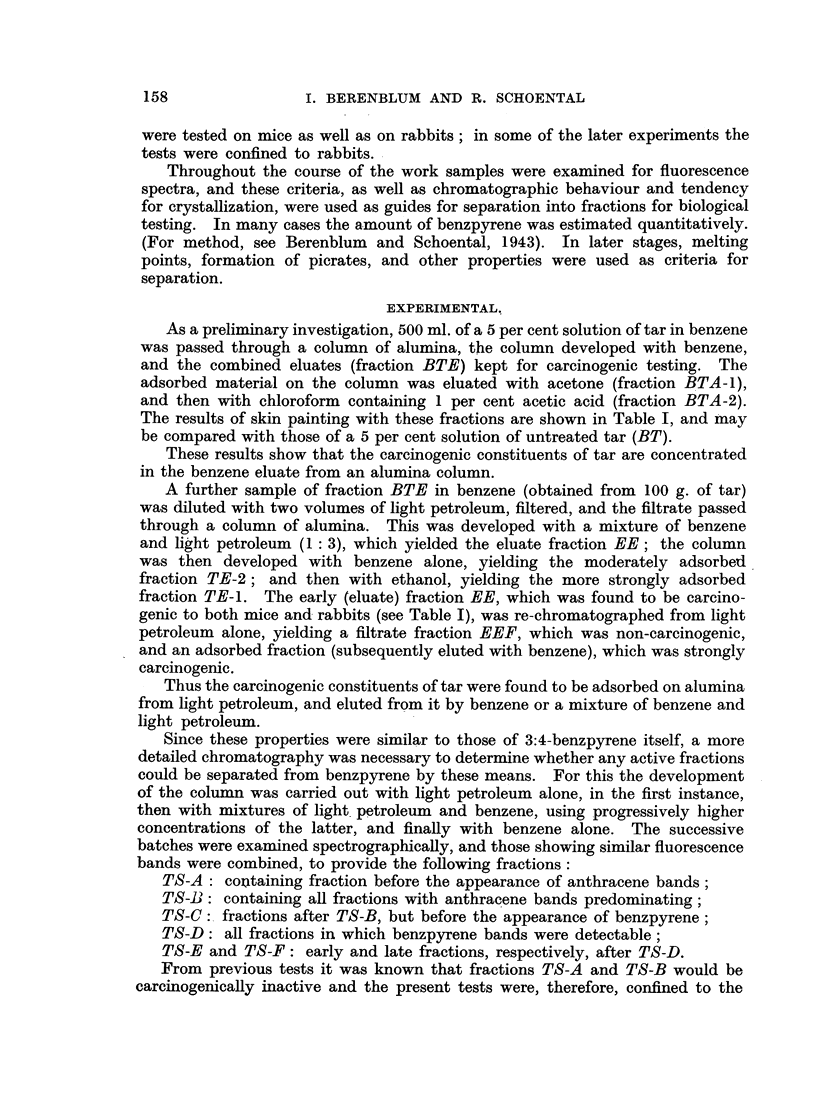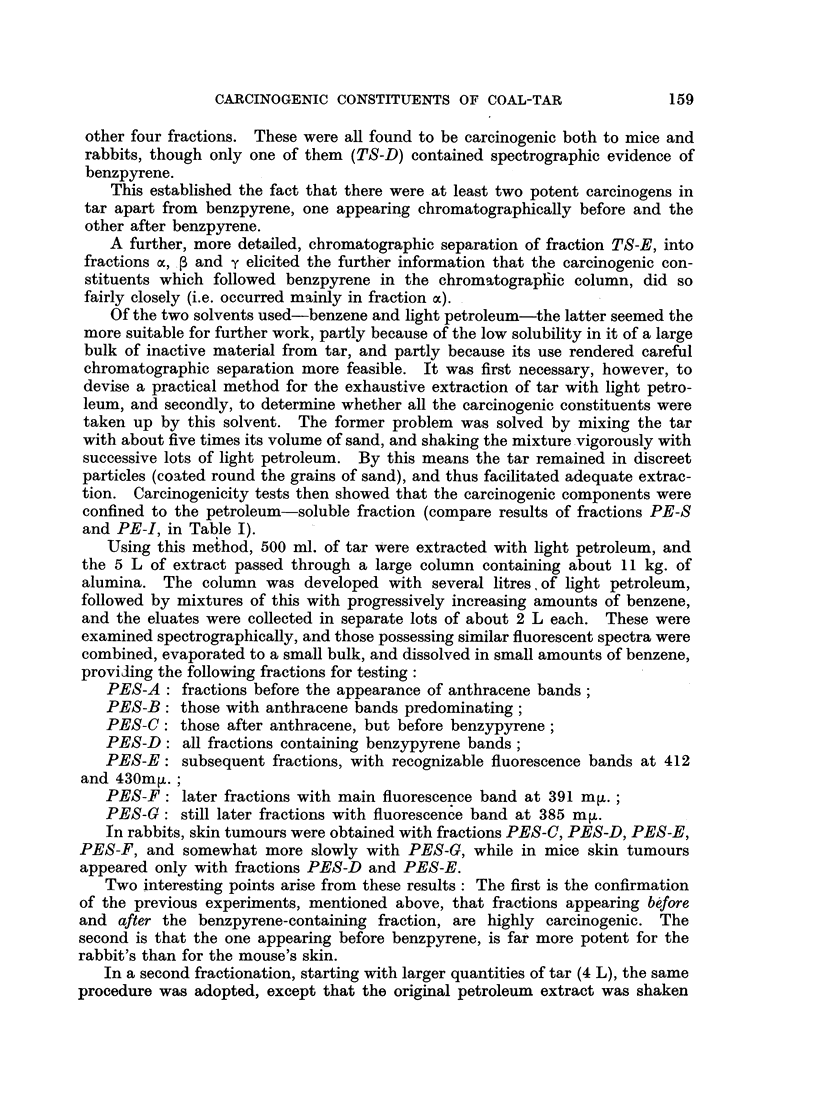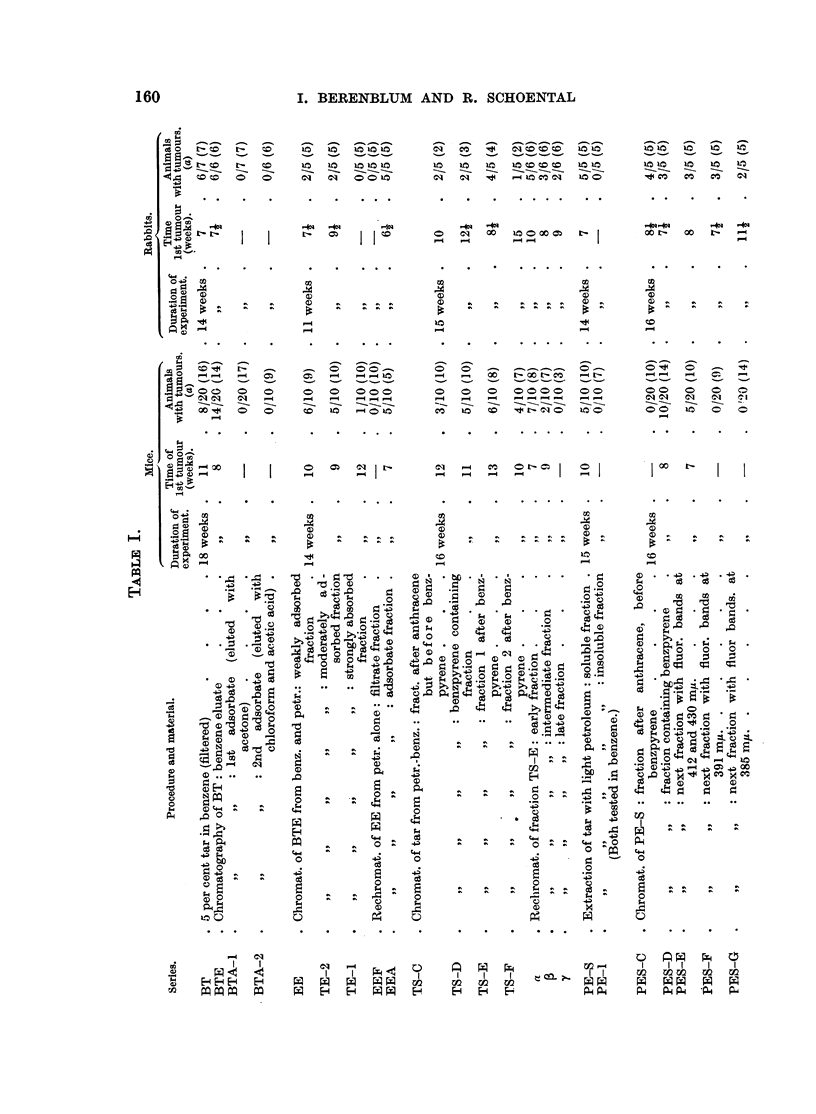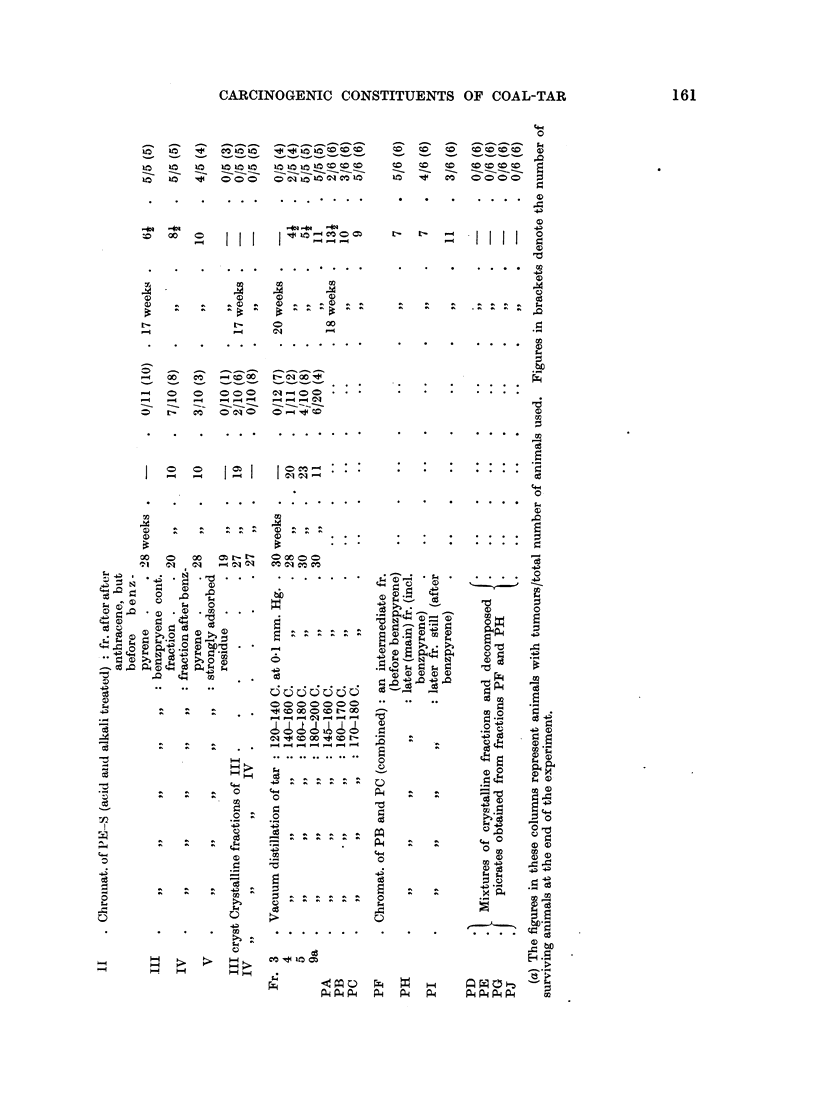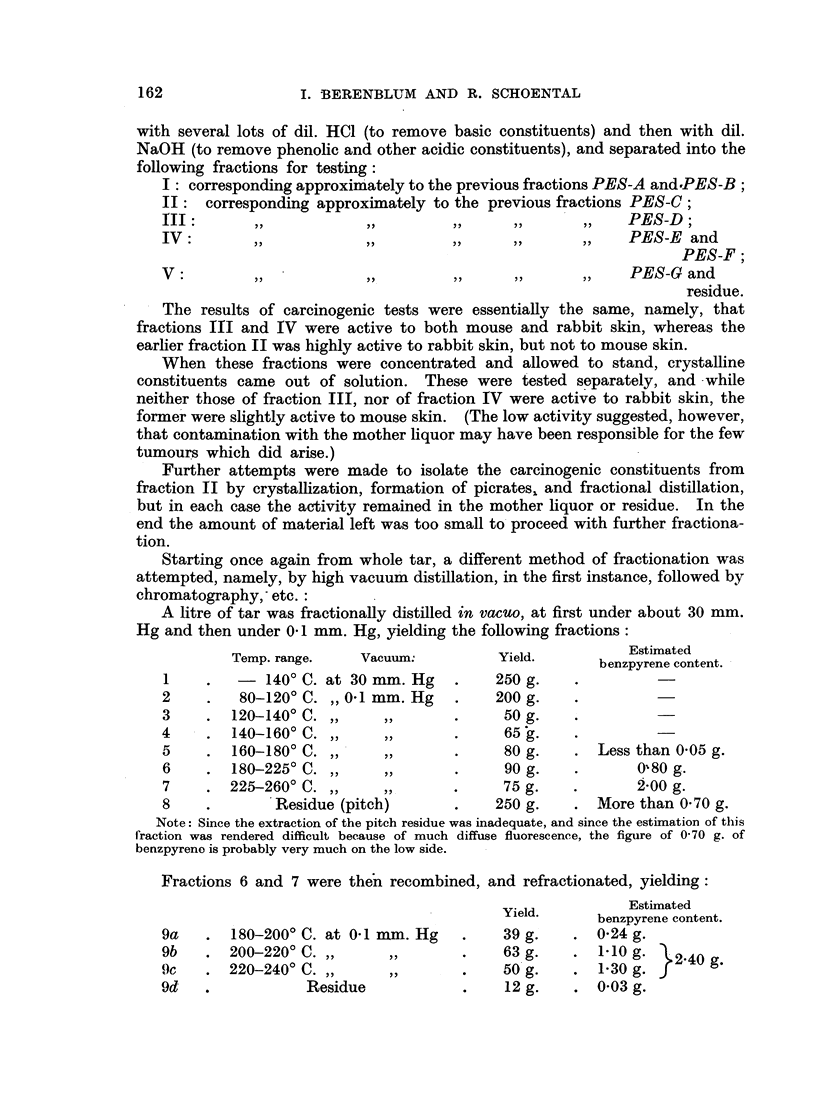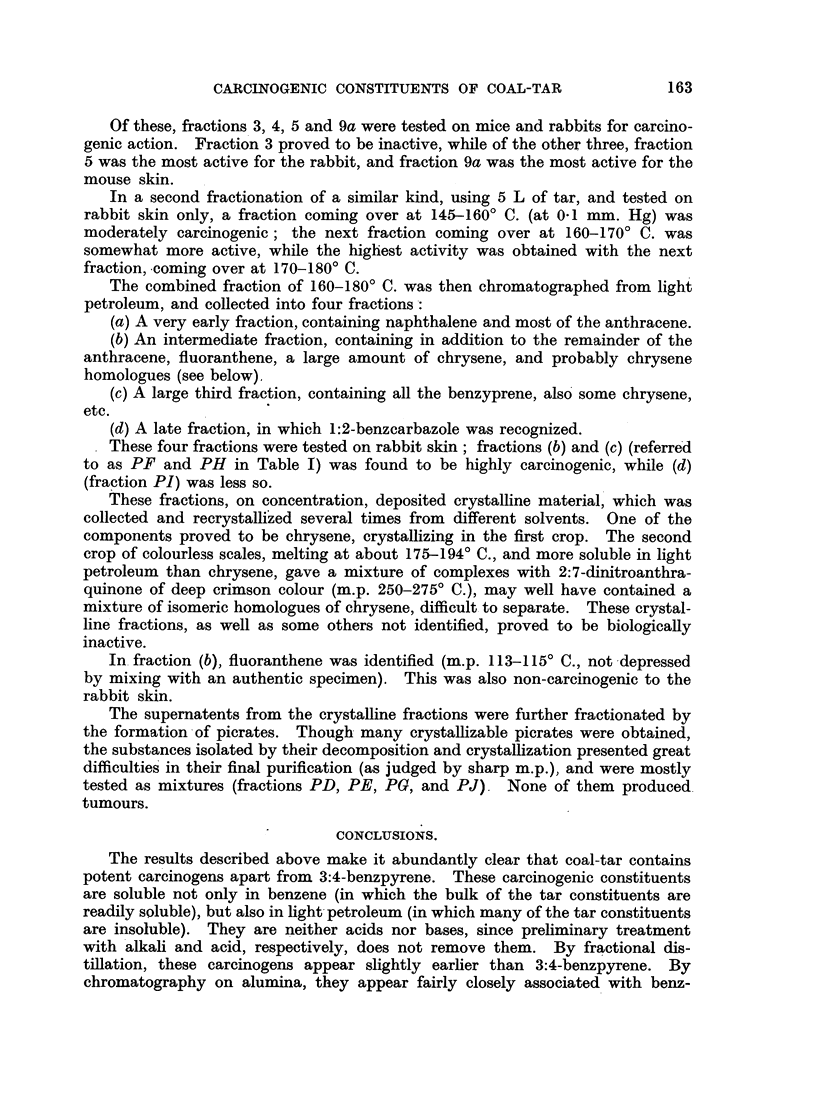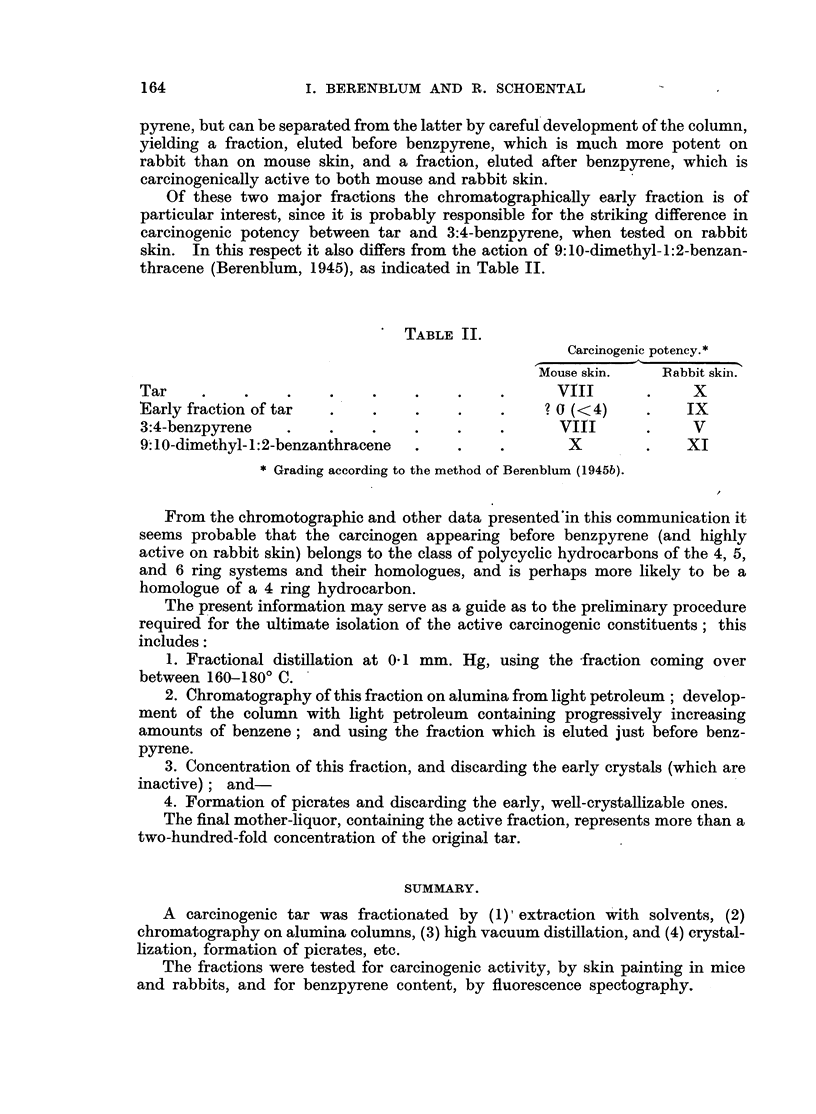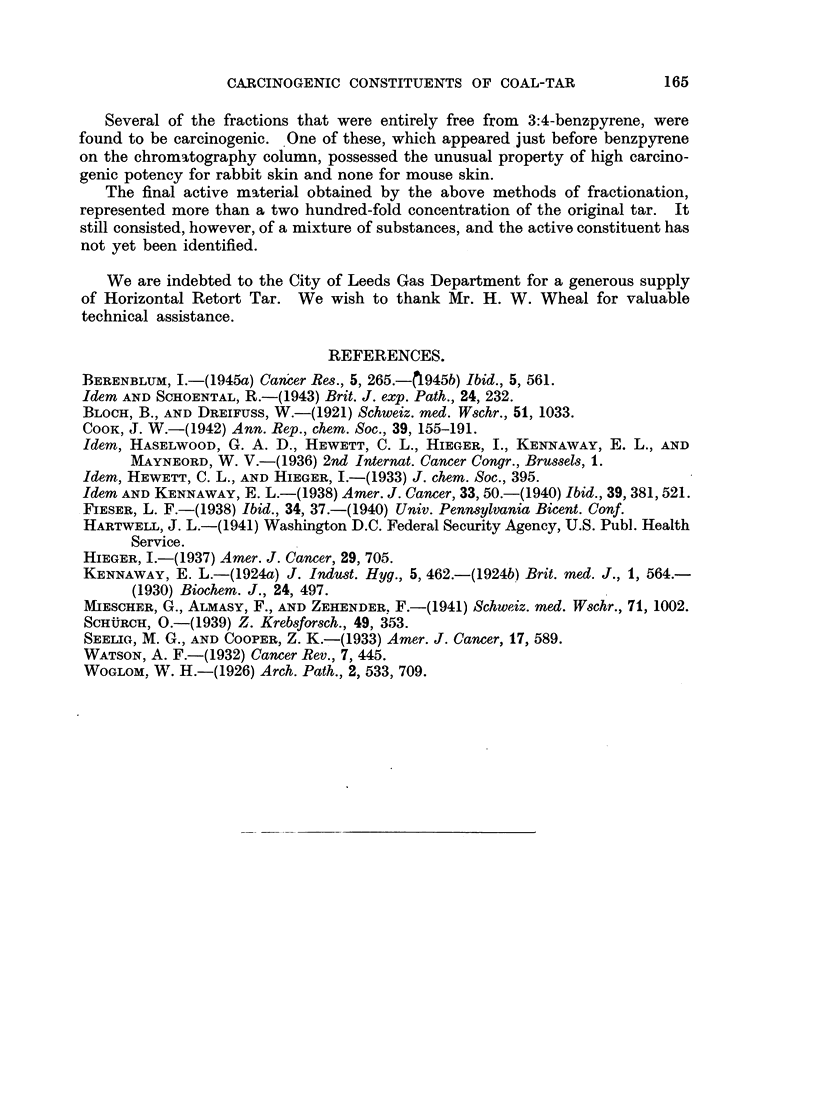# Carcinogenic Constituents of Coal-tar

**DOI:** 10.1038/bjc.1947.18

**Published:** 1947-06

**Authors:** I. Berenblum, R. Schoental


### CARCINOGENIC CONSTITUENTS OF COAL-TAR.

I. BERENBLUM AND R. SCHOENTAL.
*From the Oxford University Research Centre of the British Empire Cancer Campaign, Sir William Dunn School of Pathology, University of Oxford.*

Received for publication April 22, 1947.

THE early work of Bloch and Dreifuss (1921), of Kennaway (1924*a*, 1924*b*, 1930), and others, on the chemistry of carcinogenic tars (see reviews by Woglom, 1926 ; Watson, 1932 ; Seelig and Cooper, 1933), pointed the way to the final isolation and identification of 3:4-benzpyrene (Cook, Hewett and Hieger, 1933), and thus laid the foundation of the new phase of cancer research concerned with the synthesis of carcinogenic compounds of known constitution (see reviews by Cook, Haselwood, Hewett, Hieger, Kennaway and Mayneord, 1936 ; Cook and Kennaway, 1938, 1940 ; Fieser, 1938, 1940 ; Hartwell, 1941 ; Cook, 1942).

However, with the rapid advance in the work on the synthetic carcinogens, interest in carcinogenic tars diminished, and the previous indications that coal-tar might possibly contain potent carcinogens other than benzpyrene (Hieger, 1937), were not followed up. There has since been a growing tendency in the literature to accept benzpyrene as the only carcinogen present in tar, as exemplified by the spectrographic work of Miescher, Almasy and Zehender (1941), despite the biological evidence (Schürch, 1939 ; Berenblum, 1945*a* ; and others) of striking differences between the carcinogenic action of tar and that of benzpyrene (e.g. when tested on rabbit's skin.

The present re-investigation of carcinogenic constituents of tar, which was begun in 1941, was undertaken in order to determine whether carcinogens other than benzpyrene did, in fact, exist in tar, and if so, whether their properties were significantly different from those of the known carcinogens.

### METHODS.

A horizontal retort tar of high carcinogenic potency, obtained from the City of Leeds Gas Department, was used for this investigation. The methods of fractionation included (1) extraction with benzene, light petroleum (b.p. 60–80° C.), or mixtures of the two ; (2) chromatography, using alumina (" Aluminium Oxide for Adsorption Purposes," B.D.H. Ltd.) in columns ranging from 1–12 kg. capacity ; (3) high vacuum distillation (at 0·05–0·2 mm. Hg) ; and (4) crystallization, formation of picrates, etc.

Since the object of the biological tests was to determine in which of the fractions the carcinogens were present in high concentration, rather than to evaluate the exact potencies of the weaker fractions, relatively small groups of animals were used for each test, and the experiments only continued for a sufficient time to enable us to recognize rapidly which were the most active fractions. Consequently, in the results recorded below, failure to produce tumours does not necessarily denote *complete* absence of carcinogenic activity.

Tests for carcinogenic action were carried out by skin painting ; once weekly in mice and twice weekly in rabbits. In the early stages of the work, all fractions were tested on mice as well as on rabbits ; in some of the later experiments the tests were confined to rabbits.

Throughout the course of the work samples were examined for fluorescence spectra, and these criteria, as well as chromatographic behaviour and tendency for crystallization, were used as guides for separation into fractions for biological testing. In many cases the amount of benzpyrene was estimated quantitatively. (For method, see Berenblum and Schoental, 1943). In later stages, melting points, formation of picrates, and other properties were used as criteria for separation.

### EXPERIMENTAL.

As a preliminary investigation, 500 ml. of a 5 per cent solution of tar in benzene was passed through a column of alumina, the column developed with benzene, and the combined eluates (fraction *BTE*) kept for carcinogenic testing. The adsorbed material on the column was eluated with acetone (fraction *BTA*-1), and then with chloroform containing 1 per cent acetic acid (fraction *BTA*-2). The results of skin painting with these fractions are shown in Table I, and may be compared with those of a 5 per cent solution of untreated tar (*BT*).

These results show that the carcinogenic constituents of tar are concentrated in the benzene eluate from an alumina column.

A further sample of fraction *BTE* in benzene (obtained from 100 g. of tar) was diluted with two volumes of light petroleum, filtered, and the filtrate passed through a column of alumina. This was developed with a mixture of benzene and light petroleum (1 : 3), which yielded the eluate fraction *EE* ; the column was then developed with benzene alone, yielding the moderately adsorbed fraction *TE*-2 ; and then with ethanol, yielding the more strongly adsorbed fraction *TE*-1. The early (eluate) fraction *EE*, which was found to be carcinogenic to both mice and rabbits (see Table I), was re-chromatographed from light petroleum alone, yielding a filtrate fraction *EEF*, which was non-carcinogenic, and an adsorbed fraction (subsequently eluted with benzene), which was strongly carcinogenic.

Thus the carcinogenic constituents of tar were found to be adsorbed on alumina from light petroleum, and eluted from it by benzene or a mixture of benzene and light petroleum.

Since these properties were similar to those of 3:4-benzpyrene itself, a more detailed chromatography was necessary to determine whether any active fractions could be separated from benzpyrene by these means. For this the development of the column was carried out with light petroleum alone, in the first instance, then with mixtures of light petroleum and benzene, using progressively higher concentrations of the latter, and finally with benzene alone. The successive batches were examined spectrographically, and those showing similar fluorescence bands were combined, to provide the following fractions :

*TS-A* : containing fraction before the appearance of anthracene bands ;
*TS-B* : containing all fractions with anthracene bands predominating ;
*TS-C* : fractions after *TS-B*, but before the appearance of benzpyrene ;
*TS-D* : all fractions in which benzpyrene bands were detectable ;
*TS-E* and *TS-F* : early and late fractions, respectively, after *TS-D*.

From previous tests it was known that fractions *TS-A* and *TS-B* would be carcinogenically inactive and the present tests were, therefore, confined to the other four fractions. These were all found to be carcinogenic both to mice and rabbits, though only one of them (*TS-D*) contained spectrographic evidence of benzpyrene.

This established the fact that there were at least two potent carcinogens in tar apart from benzpyrene, one appearing chromatographically before and the other after benzpyrene.

A further, more detailed, chromatographic separation of fraction *TS-E*, into fractions $\alpha$, $\beta$ and $\gamma$ elicited the further information that the carcinogenic constituents which followed benzpyrene in the chromatographic column, did so fairly closely (i.e. occurred mainly in fraction $\alpha$).

Of the two solvents used—benzene and light petroleum—the latter seemed the more suitable for further work, partly because of the low solubility in it of a large bulk of inactive material from tar, and partly because its use rendered careful chromatographic separation more feasible. It was first necessary, however, to devise a practical method for the exhaustive extraction of tar with light petroleum, and secondly, to determine whether all the carcinogenic constituents were taken up by this solvent. The former problem was solved by mixing the tar with about five times its volume of sand, and shaking the mixture vigorously with successive lots of light petroleum. By this means the tar remained in discreet particles (coated round the grains of sand), and thus facilitated adequate extraction. Carcinogenicity tests then showed that the carcinogenic components were confined to the petroleum—soluble fraction (compare results of fractions *PE-S* and *PE-I*, in Table I).

Using this method, 500 ml. of tar were extracted with light petroleum, and the 5 L of extract passed through a large column containing about 11 kg. of alumina. The column was developed with several litres of light petroleum, followed by mixtures of this with progressively increasing amounts of benzene, and the eluates were collected in separate lots of about 2 L each. These were examined spectrographically, and those possessing similar fluorescent spectra were combined, evaporated to a small bulk, and dissolved in small amounts of benzene, providing the following fractions for testing :

*PES-A* : fractions before the appearance of anthracene bands ;

*PES-B* : those with anthracene bands predominating ;

*PES-C* : those after anthracene, but before benzypyrene ;

*PES-D* : all fractions containing benzypyrene bands ;

*PES-E* : subsequent fractions, with recognizable fluorescence bands at 412 and 430m$\mu$. ;

*PES-F* : later fractions with main fluorescence band at 391 m$\mu$. ;

*PES-G* : still later fractions with fluorescence band at 385 m$\mu$.

In rabbits, skin tumours were obtained with fractions *PES-C*, *PES-D*, *PES-E*, *PES-F*, and somewhat more slowly with *PES-G*, while in mice skin tumours appeared only with fractions *PES-D* and *PES-E*.

Two interesting points arise from these results : The first is the confirmation of the previous experiments, mentioned above, that fractions appearing *before* and *after* the benzpyrene-containing fraction, are highly carcinogenic. The second is that the one appearing before benzpyrene, is far more potent for the rabbit's than for the mouse's skin.

In a second fractionation, starting with larger quantities of tar (4 L), the same procedure was adopted, except that the original petroleum extract was shaken

### Table I.

| Series. | Procedure and material. | Mice. Duration of experiment. | Mice. Time of 1st tumour (weeks). | Mice. Animals with tumours. (a) | Rabbits. Duration of experiment. | Rabbits. Time 1st tumour (weeks). | Rabbits. Animals with tumours. (a) |
|---|---|---|---|---|---|---|---|
| BT | 5 per cent tar in benzene (filtered) | 18 weeks | 11 | 8/20 (16) | 14 weeks | 7 | 6/7 (7) |
| BTE | Chromatography of BT : benzene eluate | ,, | 8 | 14/20 (14) | ,, | 7½ | 6/6 (6) |
| BTA-1 | ,, ,, : 1st adsorbate (eluted with acetone) | ,, | — | 0/20 (17) | ,, | — | 0/7 (7) |
| BTA-2 | ,, ,, : 2nd adsorbate (eluted with chloroform and acetic acid) | ,, | — | 0/10 (9) | ,, | — | 0/6 (6) |
| EE | Chromat. of BTE from benz. and petr.: weakly adsorbed fraction | 14 weeks | 10 | 6/10 (9) | 11 weeks | 7½ | 2/5 (5) |
| TE-2 | ,, ,, ,, ,, ,, : moderately adsorbed fraction | ,, | 9 | 5/10 (10) | ,, | 9½ | 2/5 (5) |
| TE-1 | ,, ,, ,, ,, ,, : strongly absorbed fraction | ,, | 12 | 1/10 (10) | ,, | — | 0/5 (5) |
| EEF | Rechromat. of EE from petr. alone : filtrate fraction | ,, | — | 0/10 (10) | ,, | — | 0/5 (5) |
| EEA | ,, ,, ,, ,, : adsorbate fraction | ,, | 7 | 5/10 (5) | ,, | 6½ | 5/5 (5) |
| TS-C | Chromat. of tar from petr.-benz. : fract. after anthracene but before benzpyrene | 16 weeks | 12 | 3/10 (10) | 15 weeks | 10 | 2/5 (2) |
| TS-D | ,, ,, ,, ,, : benzpyrene containing fraction | ,, | 11 | 5/10 (10) | ,, | 12½ | 2/5 (3) |
| TS-E | ,, ,, ,, ,, : fraction 1 after benzpyrene | ,, | 13 | 6/10 (8) | ,, | 8½ | 4/5 (4) |
| TS-F | ,, ,, ,, ,, : fraction 2 after benzpyrene | ,, | 10 | 4/10 (7) | ,, | 15 | 1/5 (2) |
| α | Rechromat. of fraction TS-E : early fraction | ,, | 7 | 7/10 (8) | ,, | 10 | 5/6 (6) |
| β | ,, ,, ,, ,, : intermediate fraction | ,, | 9 | 2/10 (7) | ,, | 8 | 3/6 (6) |
| γ | ,, ,, ,, ,, : late fraction | ,, | — | 0/10 (3) | ,, | 9 | 2/6 (6) |
| PE-S | Extraction of tar with light petroleum : soluble fraction | 15 weeks | 10 | 5/10 (10) | 14 weeks | 7 | 5/5 (5) |
| PE-1 | ,, ,, ,, ,, ,, : insoluble fraction (Both tested in benzene.) | ,, | — | 0/10 (7) | ,, | — | 0/5 (5) |
| PES-C | Chromat. of PE-S : fraction after anthracene, before benzpyrene | 16 weeks | — | 0/20 (10) | 16 weeks | 8½ | 4/5 (5) |
| PES-D | ,, ,, : fraction containing benzpyrene | ,, | 8 | 10/20 (14) | ,, | 7½ | 3/5 (5) |
| PES-E | ,, ,, : next fraction with fluor. bands at 412 and 430 mμ. | ,, | 7 | 5/20 (10) | ,, | 8 | 3/5 (5) |
| PES-F | ,, ,, : next fraction with fluor. bands at 391 mμ. | ,, | — | 0/20 (9) | ,, | 7½ | 3/5 (5) |
| PES-G | ,, ,, : next fraction with fluor bands. at 385 mμ. | ,, | — | 0/20 (14) | ,, | 11½ | 2/5 (5) |

| | | | | | | | |
|---|---|---|---|---|---|---|---|
| II | Chromat. of PE–S (acid and alkali treated) : fr. after after anthracene, but before benzpyrene | 28 weeks | — | 0/11 (10) | 17 weeks | 6½ | 5/5 (5) |
| III | ,, ,, ,, ,, ,, : benzpryene cont. fraction | 20 ,, | 10 | 7/10 (8) | ,, | 8½ | 5/5 (5) |
| IV | ,, ,, ,, ,, ,, : fraction after benzpyrene | 28 ,, | 10 | 3/10 (3) | ,, | 10 | 4/5 (4) |
| V | ,, ,, ,, ,, ,, : strongly adsorbed residue | 19 ,, | — | 0/10 (1) | ,, | — | 0/5 (3) |
| III cryst | Crystalline fractions of III | 27 ,, | 19 | 2/10 (6) | 17 weeks | — | 0/5 (5) |
| IV ,, | ,, ,, IV | 27 ,, | — | 0/10 (8) | ,, | — | 0/5 (5) |
| Fr. 3 | Vacuum distillation of tar : 120–140 C. at 0·1 mm. Hg. | 30 weeks | — | 0/12 (7) | 20 weeks | — | 0/5 (4) |
| 4 | ,, ,, ,, : 140–160 C. ,, | 28 ,, | 20 | 1/11 (2) | ,, | 4½ | 2/5 (4) |
| 5 | ,, ,, ,, : 160–180 C. ,, | 30 ,, | 23 | 4/10 (8) | ,, | 5½ | 5/5 (5) |
| 9a | ,, ,, ,, : 180–200 C. ,, | 30 ,, | 11 | 6/20 (4) | ,, | 11 | 5/5 (5) |
| PA | ,, ,, ,, : 145–160 C. ,, | .. | .. | .. | 18 weeks | 13½ | 2/6 (6) |
| PB | ,, ,, ,, : 160–170 C. ,, | .. | .. | .. | ,, | 10 | 3/6 (6) |
| PC | ,, ,, ,, : 170–180 C. ,, | .. | .. | .. | ,, | 9 | 5/6 (6) |
| PF | Chromat. of PB and PC (combined) : an intermediate fr. (before benzpyrene) | .. | .. | .. | ,, | 7 | 5/6 (6) |
| PH | ,, ,, ,, ,, : later (main) fr. (incl. benzpyrene) | .. | .. | .. | ,, | 7 | 4/6 (6) |
| PI | ,, ,, ,, ,, : later fr. still (after benzpyrene) | .. | .. | .. | ,, | 11 | 3/6 (6) |
| PD | Mixtures of crystalline fractions and decomposed picrates obtained from fractions PF and PH | .. | .. | .. | ,, | — | 0/6 (6) |
| PE | | .. | .. | .. | ,, | — | 0/6 (6) |
| PG | | .. | .. | .. | ,, | — | 0/6 (6) |
| PJ | | .. | .. | .. | ,, | — | 0/6 (6) |

(*a*) The figures in these columns represent animals with tumours/total number of animals used. Figures in brackets denote the number of surviving animals at the end of the experiment.

with several lots of dil. HCl (to remove basic constituents) and then with dil. NaOH (to remove phenolic and other acidic constituents), and separated into the following fractions for testing :

I : corresponding approximately to the previous fractions *PES-A* and *PES-B* ;
II : corresponding approximately to the previous fractions *PES-C* ;
III : ,, ,, ,, ,, ,, *PES-D* ;
IV : ,, ,, ,, ,, ,, *PES-E* and *PES-F* ;
V : ,, ,, ,, ,, ,, *PES-G* and residue.

The results of carcinogenic tests were essentially the same, namely, that fractions III and IV were active to both mouse and rabbit skin, whereas the earlier fraction II was highly active to rabbit skin, but not to mouse skin.

When these fractions were concentrated and allowed to stand, crystalline constituents came out of solution. These were tested separately, and while neither those of fraction III, nor of fraction IV were active to rabbit skin, the former were slightly active to mouse skin. (The low activity suggested, however, that contamination with the mother liquor may have been responsible for the few tumours which did arise.)

Further attempts were made to isolate the carcinogenic constituents from fraction II by crystallization, formation of picrates, and fractional distillation, but in each case the activity remained in the mother liquor or residue. In the end the amount of material left was too small to proceed with further fractionation.

Starting once again from whole tar, a different method of fractionation was attempted, namely, by high vacuum distillation, in the first instance, followed by chromatography, etc. :

A litre of tar was fractionally distilled *in vacuo*, at first under about 30 mm. Hg and then under 0·1 mm. Hg, yielding the following fractions :

| | Temp. range. | Vacuum. | Yield. | Estimated benzpyrene content. |
|---|---|---|---|---|
| 1 | — 140° C. | at 30 mm. Hg | 250 g. | — |
| 2 | 80–120° C. | ,, 0·1 mm. Hg | 200 g. | — |
| 3 | 120–140° C. | ,, ,, | 50 g. | — |
| 4 | 140–160° C. | ,, ,, | 65 g. | — |
| 5 | 160–180° C. | ,, ,, | 80 g. | Less than 0·05 g. |
| 6 | 180–225° C. | ,, ,, | 90 g. | 0·80 g. |
| 7 | 225–260° C. | ,, ,, | 75 g. | 2·00 g. |
| 8 | Residue (pitch) | | 250 g. | More than 0·70 g. |

Note: Since the extraction of the pitch residue was inadequate, and since the estimation of this fraction was rendered difficult because of much diffuse fluorescence, the figure of 0·70 g. of benzpyrene is probably very much on the low side.

Fractions 6 and 7 were then recombined, and refractionated, yielding :

| | | Yield. | Estimated benzpyrene content. | |
|---|---|---|---|---|
| 9*a* | 180–200° C. at 0·1 mm. Hg | 39 g. | 0·24 g. | |
| 9*b* | 200–220° C. ,, ,, | 63 g. | 1·10 g. | 2·40 g. |
| 9*c* | 220–240° C. ,, ,, | 50 g. | 1·30 g. | |
| 9*d* | Residue | 12 g. | 0·03 g. | |

Of these, fractions 3, 4, 5 and 9*a* were tested on mice and rabbits for carcinogenic action. Fraction 3 proved to be inactive, while of the other three, fraction 5 was the most active for the rabbit, and fraction 9*a* was the most active for the mouse skin.

In a second fractionation of a similar kind, using 5 L of tar, and tested on rabbit skin only, a fraction coming over at 145–160° C. (at 0·1 mm. Hg) was moderately carcinogenic ; the next fraction coming over at 160–170° C. was somewhat more active, while the highest activity was obtained with the next fraction, coming over at 170–180° C.

The combined fraction of 160–180° C. was then chromatographed from light petroleum, and collected into four fractions :

(*a*) A very early fraction, containing naphthalene and most of the anthracene.

(*b*) An intermediate fraction, containing in addition to the remainder of the anthracene, fluoranthene, a large amount of chrysene, and probably chrysene homologues (see below).

(*c*) A large third fraction, containing all the benzyprene, also some chrysene, etc.

(*d*) A late fraction, in which 1:2-benzcarbazole was recognized.

These four fractions were tested on rabbit skin ; fractions (*b*) and (*c*) (referred to as *PF* and *PH* in Table I) was found to be highly carcinogenic, while (*d*) (fraction *PI*) was less so.

These fractions, on concentration, deposited crystalline material, which was collected and recrystallized several times from different solvents. One of the components proved to be chrysene, crystallizing in the first crop. The second crop of colourless scales, melting at about 175–194° C., and more soluble in light petroleum than chrysene, gave a mixture of complexes with 2:7-dinitroanthraquinone of deep crimson colour (m.p. 250–275° C.), may well have contained a mixture of isomeric homologues of chrysene, difficult to separate. These crystalline fractions, as well as some others not identified, proved to be biologically inactive.

In fraction (*b*), fluoranthene was identified (m.p. 113–115° C., not depressed by mixing with an authentic specimen). This was also non-carcinogenic to the rabbit skin.

The supernatents from the crystalline fractions were further fractionated by the formation of picrates. Though many crystallizable picrates were obtained, the substances isolated by their decomposition and crystallization presented great difficulties in their final purification (as judged by sharp m.p.), and were mostly tested as mixtures (fractions *PD*, *PE*, *PG*, and *PJ*). None of them produced tumours.

### CONCLUSIONS.

The results described above make it abundantly clear that coal-tar contains potent carcinogens apart from 3:4-benzpyrene. These carcinogenic constituents are soluble not only in benzene (in which the bulk of the tar constituents are readily soluble), but also in light petroleum (in which many of the tar constituents are insoluble). They are neither acids nor bases, since preliminary treatment with alkali and acid, respectively, does not remove them. By fractional distillation, these carcinogens appear slightly earlier than 3:4-benzpyrene. By chromatography on alumina, they appear fairly closely associated with benzpyrene, but can be separated from the latter by careful development of the column, yielding a fraction, eluted before benzpyrene, which is much more potent on rabbit than on mouse skin, and a fraction, eluted after benzpyrene, which is carcinogenically active to both mouse and rabbit skin.

Of these two major fractions the chromatographically early fraction is of particular interest, since it is probably responsible for the striking difference in carcinogenic potency between tar and 3:4-benzpyrene, when tested on rabbit skin. In this respect it also differs from the action of 9:10-dimethyl-1:2-benzanthracene (Berenblum, 1945), as indicated in Table II.

TABLE II.

| | Carcinogenic potency.* | |
|---|---|---|
| | Mouse skin. | Rabbit skin. |
| Tar | VIII | X |
| Early fraction of tar | ? 0 (<4) | IX |
| 3:4-benzpyrene | VIII | V |
| 9:10-dimethyl-1:2-benzanthracene | X | XI |

* Grading according to the method of Berenblum (1945*b*).

From the chromotographic and other data presented in this communication it seems probable that the carcinogen appearing before benzpyrene (and highly active on rabbit skin) belongs to the class of polycyclic hydrocarbons of the 4, 5, and 6 ring systems and their homologues, and is perhaps more likely to be a homologue of a 4 ring hydrocarbon.

The present information may serve as a guide as to the preliminary procedure required for the ultimate isolation of the active carcinogenic constituents ; this includes :

1. Fractional distillation at 0·1 mm. Hg, using the fraction coming over between 160–180° C.
2. Chromatography of this fraction on alumina from light petroleum ; development of the column with light petroleum containing progressively increasing amounts of benzene ; and using the fraction which is eluted just before benzpyrene.
3. Concentration of this fraction, and discarding the early crystals (which are inactive) ; and—
4. Formation of picrates and discarding the early, well-crystallizable ones.

The final mother-liquor, containing the active fraction, represents more than a two-hundred-fold concentration of the original tar.

### SUMMARY.

A carcinogenic tar was fractionated by (1) extraction with solvents, (2) chromatography on alumina columns, (3) high vacuum distillation, and (4) crystallization, formation of picrates, etc.

The fractions were tested for carcinogenic activity, by skin painting in mice and rabbits, and for benzpyrene content, by fluorescence spectography.

Several of the fractions that were entirely free from 3:4-benzpyrene, were found to be carcinogenic. One of these, which appeared just before benzpyrene on the chromatography column, possessed the unusual property of high carcinogenic potency for rabbit skin and none for mouse skin.

The final active material obtained by the above methods of fractionation, represented more than a two hundred-fold concentration of the original tar. It still consisted, however, of a mixture of substances, and the active constituent has not yet been identified.

We are indebted to the City of Leeds Gas Department for a generous supply of Horizontal Retort Tar. We wish to thank Mr. H. W. Wheal for valuable technical assistance.

### REFERENCES.

Berenblum, I.—(1945*a*) *Cancer Res.*, **5**, 265.—(1945*b*) *Ibid.*, **5**, 561.

*Idem* and Schoental, R.—(1943) *Brit. J. exp. Path.*, **24**, 232.

Bloch, B., and Dreifuss, W.—(1921) *Schweiz. med. Wschr.*, **51**, 1033.

Cook, J. W.—(1942) *Ann. Rep., chem. Soc.*, **39**, 155–191.

*Idem*, Haselwood, G. A. D., Hewett, C. L., Hieger, I., Kennaway, E. L., and Mayneord, W. V.—(1936) *2nd Internat. Cancer Congr., Brussels*, **1**.

*Idem*, Hewett, C. L., and Hieger, I.—(1933) *J. chem. Soc.*, 395.

*Idem* and Kennaway, E. L.—(1938) *Amer. J. Cancer*, **33**, 50.—(1940) *Ibid.*, **39**, 381, 521.

Fieser, L. F.—(1938) *Ibid.*, **34**, 37.—(1940) *Univ. Pennsylvania Bicent. Conf.*

Hartwell, J. L.—(1941) Washington D.C. Federal Security Agency, U.S. Publ. Health Service.

Hieger, I.—(1937) *Amer. J. Cancer*, **29**, 705.

Kennaway, E. L.—(1924*a*) *J. Indust. Hyg.*, **5**, 462.—(1924*b*) *Brit. med. J.*, **1**, 564.—(1930) *Biochem. J.*, **24**, 497.

Miescher, G., Almasy, F., and Zehender, F.—(1941) *Schweiz. med. Wschr.*, **71**, 1002.

Schürch, O.—(1939) *Z. Krebsforsch.*, **49**, 353.

Seelig, M. G., and Cooper, Z. K.—(1933) *Amer. J. Cancer*, **17**, 589.

Watson, A. F.—(1932) *Cancer Rev.*, **7**, 445.

Woglom, W. H.—(1926) *Arch. Path.*, **2**, 533, 709.